# Prognostic significance of ASXL1 mutations in acute myeloid leukemia: A systematic review and meta-analysis

**DOI:** 10.22088/cjim.15.2.202

**Published:** 2024

**Authors:** Maryam Sheikhi, Mehrdad Rostami, Gordon Ferns, Hossein Ayatollahi, Payam Siyadat, Yasamin Ayatollahi, Zahra Khoshnegah

**Affiliations:** 1 Cancer Molecular Pathology Research Center, Mashhad University of Medical Sciences, Mashhad, Iran; 2 Departments of Hematology and Blood Banking, Faculty of Allied Medicine, Iran University of Medical Sciences, Tehran, Iran; 3 Division of Medical Education, Brighton and Sussex Medical School, Brighton, United Kingdom; 4 Blood Transfusion Research Center, High Institute for Research and Education in Transfusion Medicine, Tehran, Iran; 5 Department of Laboratory Hematology and Blood Banking, Faculty of Allied Medicine, Kerman University of Medical Sciences, Kerman, Iran

**Keywords:** Acute myeloid leukemia, Prognosis, Additional sex comb-like 1, ASXL1, ASXL1 mutation.

## Abstract

**Background:**

Although genetic mutations in additional sex-combs-like 1 (ASXL1) are prevalent in acute myeloid leukemia (AML), their exact impact on the AML prognosis remains uncertain. Hence, the present article was carried out to explore the prognostic importance of ASXL1 mutations in AML.

**Methods:**

We thoroughly searched electronic scientific databases to find eligible papers. Twenty-seven studies with an overall number of 8,953 participants were selected for the current systematic review. The hazard ratio (HR) and 95% confidence interval (CI) for overall survival (OS), event-free survival (EFS), and relapse-free survival (RFS) were extracted from all studies with multivariate or univariate analysis. Pooled HRs and p-values were also calculated as a part of our work.

**Results:**

The pooled HR for OS in multivariable analysis indicated that ASXL1 significantly diminished survival in AML patients (pooled HR: 1.67; 95% CI: 1.342-2.091).

**Conclusions:**

ASXL1 mutations may confer a poor prognosis in AML. Hence, they may be regarded as potential prognostic factors. However, more detailed studies with different ASXL1 mutations are suggested to shed light on this issue.

Acute myeloid leukemia (AML), a type of blood cancer, results from the inhibition of myeloid maturation and substantial genetic changes in hematopoietic stem cells (1, 2). AML usually affects patients more than 65 years old (3). Cytogenetic and molecular abnormalities have a central role in AML pathogenesis. Although previous studies have shown that some forms of AML, such as core-binding factor AML (CBF-AML), exhibit favorable outcomes, there are AML subtypes leading to poor survival (4). Even with outstanding improvements in our perception of the genetic landscape of AML, the impacts of several important mutations, such as FLT3-ITD, CEBPA, and ASXL1, still need to be fully understood. However, they may be suggested as potential factors for risk stratification and therapeutic decisions, especially when combined with other chromosomal abnormalities (5-7).

ASXL1, found on chromosome 20q11, is suggested to have epigenetic modulatory roles. Most ASXL1 mutations are located on the exon 12, typically as frameshift or nonsense mutations (8, 9) that have been frequently detected in AML. Although their exact role in hematologic malignancies has not been fully elucidated, several studies show that ASXL1 functions as a tumor-suppressor gene (10, 11).

ASXL1 somatic mutations have been previously suggested as potential markers of poor prognosis. However, their prevalence varies in different diseases, ranging from nearly half of the patients with chronic myelomonocytic leukemia (CMML) to approximately 10% in AML (30% in secondary AML versus 6.5% in de-novo AML) and 16% in myelodysplastic syndromes (MDS) (10, 11). Moreover, recent animal studies have demonstrated that mutant ASXL1 may lead to impaired hematopoiesis and myeloid transformation through alterations in histone markers (12). Since a better understanding of the roles of ASXL1 in hematopoietic malignancies seems essential, the current paper focuses on evaluating the influence of ASXL1 mutations on the survival of cases suffering from AML.

## Methods

**Search protocol:** A rigorous search was carried out in three scientific databases, including PubMed, Scopus, and Web of Knowledge, to obtain relevant studies reporting sufficient data about the influence of ASXL mutations on the survival of AML patients until 14 February 2021. We utilized the following keywords in our search: “Acute Myeloid Leukemia” OR “Acute Myelocytic Leukemia” OR “Acute Myeloblastic Leukemia” OR “AML” AND “ASXL1” OR “Additional Sex Combs Like 1 Protein”.

**Inclusion and exclusion criteria:** The inclusion criteria were set as printed articles in English until 14 February 2021, reporting Hazard ratios (HR) and 95% confidence intervals (CI) for ASXL1 mutations in patients with AML or any other reliable data by which HR and 95% CI could be calculated. Studies with insufficient data, reviews, case reports, letters, conference articles, in-vitro studies, animal research, and articles in languages except for English were eliminated.

**Data Extraction and Quality Assessment:** Two reviewers (MR and ZKH) independently inspected the studies. They gathered all the necessary information, including the first author’s name, year of publication, journal, country, number of participants, diagnosis criteria for the classification of AML, patients’ characteristics (e.g., age, sex, median WBC count), rate of ASXL1 mutations, and HRs with their 95% CI for overall survival (OS), event-free survival (EFS), and relapse-free survival (RFS). A third reviewer (MSH) was consulted to resolve any discrepancies. Utilizing the Newcastle-Ottawa Scale (NOS), we evaluated the quality of all qualified studies. According to the NOS tool, the quality score of studies could be up to 9 points. Qualified articles for the present meta-analysis showed a score between 5-8. 

**Statistical Analysis:** We utilized the comprehensive meta-analysis Version 2 (CMA 2) software for statistical analysis. Employing pooled HRs and 95% CI, we assessed the effect of ASXL1 mutations on OS, RFS, and EFS of AML patients. The I^2^ statistic was used as a suitable indicator for estimating heterogeneity. We defined I^2^ < 25% as no heterogeneity, I^2^=20-50% as moderate heterogeneity, and I^2^>50% as high heterogeneity. Two statistical models, including the random-effect model for high heterogeneity and the fixed-effect model for low/moderate heterogeneity, were applied in our meta-analysis. To demonstrate publication bias accurately, we used different methods, such as funnel plots and Begg’s and Egger’s tests. Moreover, pooled HRs>1 indicated a significantly unfavorable prognosis. We considered a p-value<0.05 as significant. 

## Results

**Figure 1:**
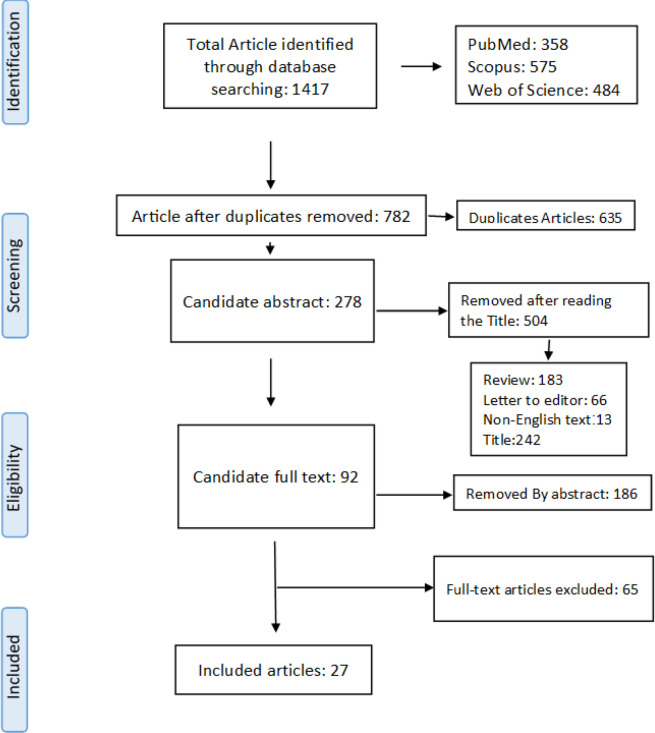
Flow diagram of the selection process in the systematic Review

**Study selection and Characteristics:** The process flow diagram of study screening for ASXL1 is shown in figure 1. Our initial search retrieved 1,417 articles, of which 358, 575, and 484 studies were obtained from PubMed, Scopus, and Web of Science databases. After eliminating duplicate papers, 782 articles were reviewed, and letters to the editors, review articles, case reports, papers with non-English languages, and studies with irrelevant titles or inadequate data (504 papers) were eliminated. After the initial screening of abstracts, we excluded 186 of the 278 remaining papers. Eventually, following careful inspection of complete texts, 27 papers were eligible for systematic analysis, and all these 27 studies (with 8,953 participants) also met the criteria for our meta-analysis (table 1). All included papers were published over the period from 2011 to 2021. Of those, 12 studies were from Europe, seven studies were from the United States, and eight papers were from Asia. Also, leukemia diagnosis in all eligible studies was based on the World Health Organization (WHO) or French-American-British (FAB) criteria. As shown in table 1, all included participants were AML cases, including primary and secondary AML, AML with myelodysplasia-related changes (AML-MRC), and AML with normal cytogenetics.

**Table 1:** Summary of included studies

No.	Authors	year	Region	Patients	Type of diseases	Criteria	Age*1 ASXL1mutant ASXL1wt	Sex (M/F)* ASXL1mutant ASXL1wt	Rate of ASXL1 Mutation (%)	methods	Ref
1	Pratcorona M	2012	Netherlands	882	AML (mix)	FAB	54 (15-74) 47 (15-77)	27 /19 419 /417	46 (5%)	direct sequencing	(54)
2	Prats-Martín C	2020	Spain	61	AML-MRC	WHO	73.7(35-89)	35/26	19 (31%)	direct sequencing & NGS	(20)
3	Chou w	2010	Taiwan	501	de novo AML	FAB	66 49	39/15 246/201	54(10.8%)	direct sequencing	(25)
4	Sasaki K multivariant	2019	USA	421	AML (mix)	WHO	69 (17.1-91.8)	245/176	71(17%)	NGS	(55)
5	Höllein A	2018	Germany	134	AML (mix)	FAB	51 (18-83)	60/74	10(11%)	qPCR	(56)
6	Nazha A	2016	USA	468	pAML & sAML	WHO	64 (18–100)	268/200	16.3% in pAML 2.8% in sAML	direct sequencing	(57)
7	El-Sharkawi D	2014	UK	367	pAML & sAML	FAB	61.5 (19–74) 51 (16–80)	20/12 160/175	32(8.7%)	direct sequencing	(14)
8	Grossmann V	2012	Germany	1000	AML (mix)	WHO	66.8 (3.4-100.4)	537/463	159(15.9%)	direct sequencing & NGS	(17)
9	Krauth M-T	2014	Germany	139	t(8;21) positive AML	FAB	53.3 (18.6–83.8)	74/65	16 (11.5%)	qPCR	(58)
10	Schnittger S	2012	Germany	740	AML (mix)	WHO, FAB	71.8(9.4) 61.8(14.9)	93/34 302/311	127 (17.2%)	direct sequencing	(45)
11	Klaus H. Metzeler	2011	USA	220	CN-AML	FAB	68(61-83) 69 (60-82)	24/14 85/97	38(8.98%)	Microarray	(18)
12	Parkin B	2015	USA	156	non-M3 AML	FAB	67	NR	26% dnAML sAML1 13%	NGS	(59)
13	Allan JA	2018	USA	58	New AML	NR	73 (56-87)	33/25	27%	NGS	(60)
14	Devillier R	2015	France	125	AML-MRC	FAB	71(18-90)	NR	26(21%)	direct sequencing	(38)
15	Guopan U	2019	China	64	AML1ETOpositive AML	WHO	27.5 (265)	39/25	10 (15.6%)	NGS	(16)
16	Paschka P	2015	Germany	1696	AML (mix)	WHO	53 (36-61) 48 (16-61(	63/40 805/788	103 (6.1%)	direct sequencing	(11)
17	Rothenberg-Thurley M	2017	Germany	129	AML (mix)	WHO	54(20-80)	62/64	13 (10%)	direct sequencing	(61)
18	Saygin C	2018	USA	148	AML (mix)	WHO	58.5 (24–75)	80/68	11%	NGS	(22)
19	Stengel A	2017	Germany	467	AML (mix)	WHO	72(18 – 92)	296/171	36%	Array CGH	(23)
20	Renneville A	2013	France	191	CN-AML	WHO	62(50–70)	NR	17 (8.9%)	SNP-array karyotyping	(21)
21	YU GP	2019	China	64	AML1ETOpositive AML	FAB	27.5 (265)	39/25	10 (15.6%)	direct sequencing & NGS	(62)
22	Zong X	2016	China	78	AML patients with trisomy 8	FAB	61 (34–79) 40 (12–84)	10/5 30/33	15(10.2%)	qPCR	(24)
23	WangR-Q	2020	China	171	AML (mix)	FAB	48 (19-88)	93/78	<10%	NGS	(63)
24	Lin Y	2020	China	156	non-M3 AML	FAB	49 (20–75)	12/10	22 (17%)	NGS	(64)
25	Salmoiraghi S	2020	USA	221	New AML	WHO	52.5 (19.8–74.8)	102/119	9%	NGS	(15)
26	Ni J	2020	China	92	non-M3 AML	FAB	60-75	52/40	20%	NGS	(19)
27	Chen X	2021	China	204	non-M3 AML	FAB/WHO	51.5 (20–86)	103 /101	37(18.1%)	NGS	(13)

No, Number; M, Male; F, Female; wt, Wild Type; FAB, French-American-British; WHO, Word Health Organization; NR, Not Reported ; Ref, reference; AML, Acute Myeloid Leukemia; AML-MRC, Acute Myeloid Leukemia with myelodysplasia-related changes; pAML, primary Acute Myeloid Leukemia; sAML, secondary Acute Myeloid Leukemia; CN-AML, Cytogenetically Normal Acute Myeloid Leukemia; qPCR, quantity polymerase chain reaction; NGS, Next-Generation Sequencing; Array CGH, Array Comparative Genomic Hybridization; SNP Array, Single Nucleotide Polymorphism Array.

* Data is in the mutation and wt groups or only the mutation group is reported.

1. Data is reported on the mean (IQR) or mean.

**Figure 2:**
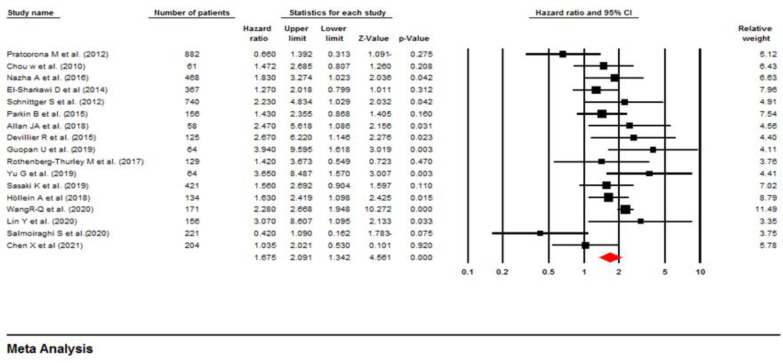
Forest plots of the HRs and 95% CI for OS in AML patients (Multivariable analysis)

**Figure 3:**
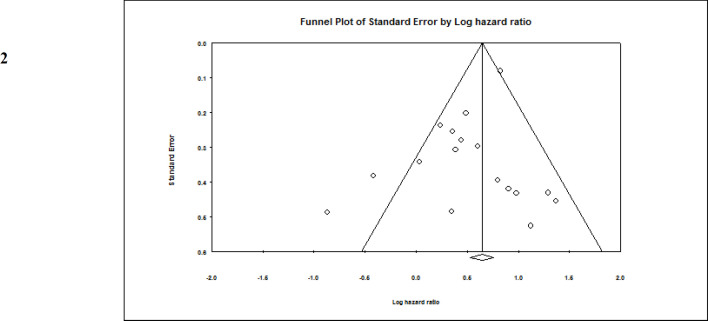
Funnel plot of the prognostic significance of ASXL1 mutation in AML patients (Multivariable analysis)

**Figure 4:**
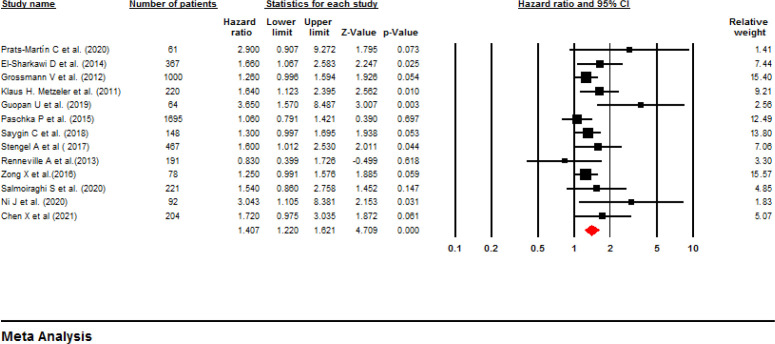
Forest plots of the HRs and 95% CI for OS in AML patients (Univariable analysis)

**Figure 5:**
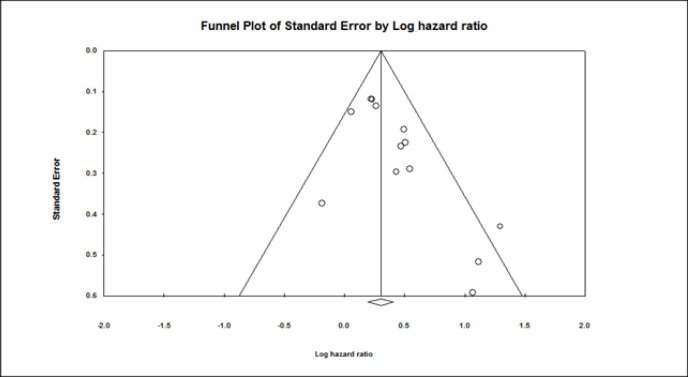
Funnel plot of the prognostic significance of ASXL1 mutation in AML patients (Univariable analysis)

**Influence of ASXL1 mutations on overall survival:** All included studies employed a multivariate or univariate analysis to evaluate the influence of ASXL1 mutations on the OS of patients with AML. Seventeen studies with 4,421 patients applied multivariable analysis with high heterogeneity (I^2^=58.8%, P=0.001). A meta-analysis of these 17 studies showed that the ASXL1 mutations were significantly correlated with a decreased OS (pooled HR:1.67; 95% CI: 1.34–2.09; P=0.000). (figure 2). Begg’s and Egger’s test results did not indicate significant bias (P=0.387 and P=0.140, respectively). The funnel plot for assessing publication bias is shown in figure 3. Furthermore, 13 articles with a total of 4,808 patients applied univariable analysis (11, 13-24). The fixed-effect model showed pooled HR for OS as 1.35 (95% CI: 1.21–1.50, P=0.000), with moderate heterogeneity (I^2^=32.94%, P=0.12) (figure 4). Consistent with multivariable analysis, Begg’s and Egger’s tests revealed no evidence of meaningful bias in univariable analysis (p-values: 0.058 and 0.011, respectively). In addition, figure 5 depicts a funnel plot of these studies.

**Figure 6:**
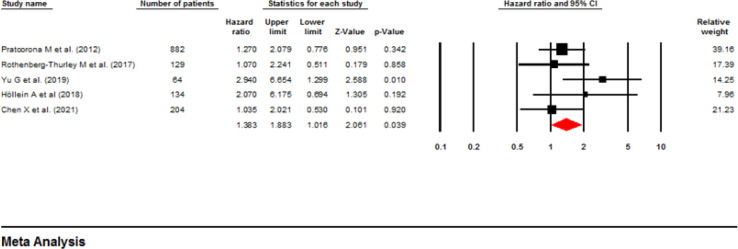
Forest plots of the HRs and 95% CI for RFS in AML patients (Multivariable analysis)

**Figure 7:**
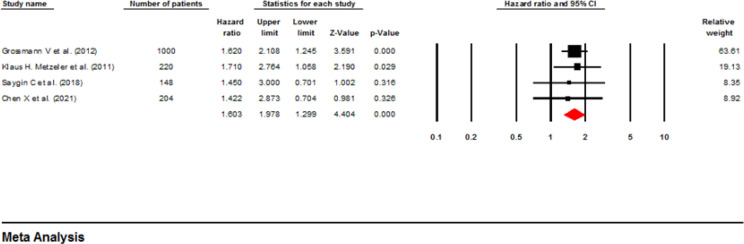
Forest plots of the HRs and 95% CI for RFS in AML patients (Univariable analysis)

**Influence of ASXL1 mutations on relapse-free survival:** Five studies comprising 1,413 patients used multivariable analysis to discover any potential correlation between ASXL1 mutations and the RFS of AML cases (13-17). The HR was calculated as 1.38 (95% CI: 1.01–1.88, P=0.039). As depicted in figure 6, a low heterogeneity was seen for these studies (I^2^ =21.5%, P=0.19). On the contrary, four articles (13, 18-20) with a total of 1,572 cases utilized univariable analysis without heterogeneity (I^2^ =00%, P=0.967). The pooled HR was 1.60 (95% CI: 1.30-1.98, P=0.000), showing a significantly shorter RFS (figure 7).

**Figure 8:**
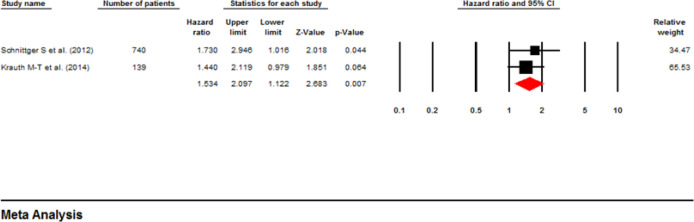
Forest plots of the HRs and 95% CI for EFS in AML patients (Multivariable analysis)

**Figure 9:**
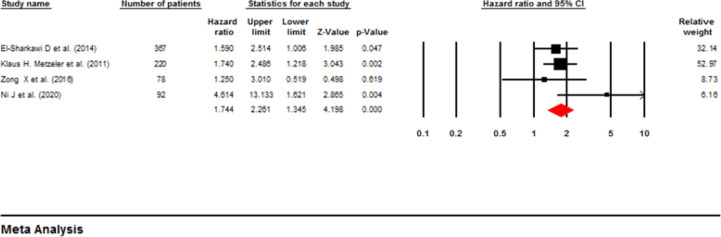
Forest plots of the HRs and 95% CI for EFS in AML patients (Univariable analysis)

**Influence of ASXL1 mutations on event-Free Survival:** Two investigations comprising 879 patients used a multivariable analysis to determine the possible correlation between ASXL1 mutations and EFS in AML patients (21, 22). Based on the fixed-effect analysis, the ASXL1 mutations significantly decreased the EFS (pooled HR: 1.53; 95% CI: 1.12-2.09; P=0.007) without heterogeneity (I^2^=00%, P=0.585) (figure. 8). On the other hand, four papers with 757 patients utilized univariable analysis (14, 18, 19, 24). The pooled HR with fixed-effect analysis was calculated as 1.74 (95% CI: 1.34-2.26, P=0.000), with moderate heterogeneity (I^2^ =25.6%, P=0.26), highlighting a reduced EFS rate in patients with AML and mutant ASXL1 (figure. 9) (19, 23-25). In addition, table 2 represents a summary of the meta-analysis, and table 3 shows Begg’s and Egger’s test results for RFS and EFS.

**Table 2:** Meta-analysis of the prognostic significance of ASXL1 mutations in AML patients

Parameters	No. studies	No. patients	Heterogeneity				Model	Meta-analysis		
			I^2^	P	T-au^2^	T-au		HR（95%CI）	Z	P
OS										
Multivariable	17	4421	58.8	0.001	0.10	0.32	Random	1.67(1.34-2.09)	4.561	0.000
Univariable	13	4808	32.94	0.12	0.02	0.14	Fixed	1.35(1.21-1.50)	5.63	0.000
RFS										
Multivariable	5	1413	21.5	0.278	0.03	0.19	Fixed	1.38(1.01-1.88)	2.06	0.039
Univariable	4	1572	0.00	0.967	0.00	0.00	Fixed	1.60(1.30-1.98)	4.40	0.000
EFS										
Multivariable	2	879	0.00	0.585	0.00	0.00	Fixed	1.53(1.12-2.09)	2.68	0.007
Univariable	4	757	25.6	0.26	0.03	0.17	Fixed	1.74(1.34-2.26)	4.2	0.000

No, Number; HR, Hazard ratio, OS, Overall Survival; RFS, Relapse-Free Survival; EFS, Event-Free Survival.

**Table 3:** Assessment of publications̛̛ bias by the Egger’s and the Begg’s test

Group	OS-M	OS-U	RFS-M	RFS-U	EFS-U
Egger’s test (P-value)	0.140	0.011	0.369	0.376	0.574
Begg’s test (P-value)	0.387	0.058	0.462	0.734	1

OS, Overall Survival; RFS, Relapse-Free Survival; EFS, Event-Free Survival; M, Multivariable; U, Univariable.

**Figure 10:**
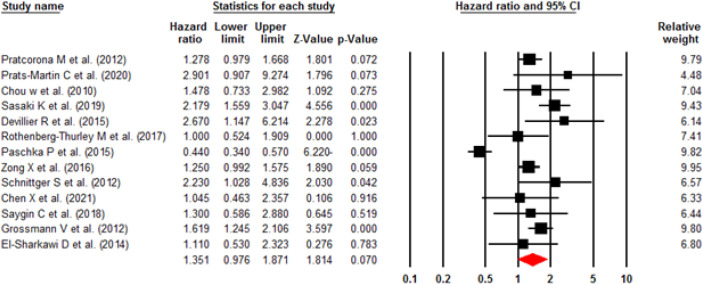
Forest plots of the HRs and 95% CI for OS in AML patients with intermediate-risk cytogenetic

**Figure 11:**
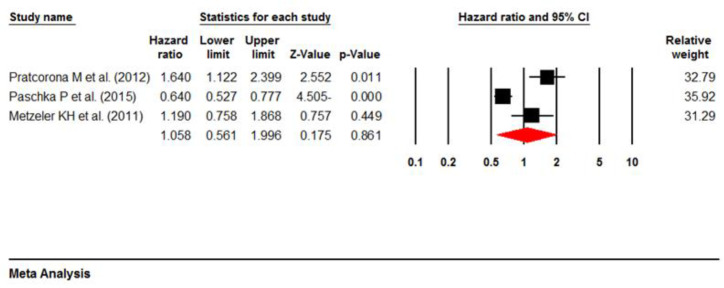
Forest plots of the HRs and 95% CI for OS in AML patients with intermediate-risk cytogenetic+NPM1 mutation

**ASXL1 prognosis in intermediate-risk cytogenetic AML and NPM1 mutations:** For assessing the impacts of intermediate-risk cytogenetics and NPM1 mutations on the prognosis of ASXL1 mutations, we initially selected studies evaluating OS in AML patients with an intermediate-risk cytogenetic profile. Based on our findings, thirteen studies comprising 6352 participants evaluated HRs in AML cases with intermediate-risk cytogenetics without NPM1 mutations (11, 13, 15, 16, 18, 20, 21, 23, 25-29). Through the random-effects model, we calculated the pooled HR as 1.35(95% CI: 0.97–1.87, P=0.07), with high heterogeneity (I^2^ =85.75%, P=0.00) (figure10). Secondly, three articles (with 2798 patients) reporting HRs for AML patients with both intermediate-risk cytogenetic profile and NPM1 mutations were analyzed (11, 15, 19). According to the random-effects model, the pooled HR for these three studies was 1.05(95% CI: 0.56–1.99, P=0.86) (figure 11), with high heterogeneity (I^2^=90.76%, P=0.00). Besides, table 4 demonstrates a summary of the results. 

**Table 4:** Meta-analysis of the prognostic significance of ASXL1 mutation in AML patients with intermediate-risk cytogenetic versus intermediate-risk cytogenetic + NPM1 mutation for Overall survival (OS)

Group	No. studies	No. patients	Heterogeneity		Model	Meta-analysis			Publication bias	
			I^2^	P		HR (95%CI）	Z	P	Egger’s test (P-value)	Begg’s test (P-value)
Intermediate-risk cytogenetic	13	6352	85.75	0.00	Random	1.35(0.97-1.87)	1.814	0.07	0.38	0.67
Intermediate-risk cytogenetic + *NPM1* mutation	3	2798	90.76	0.00	Random	1.05(0.56-1.99)	0.175	0.861	0.27	1

Abbreviation: No, Number; HR, Hazard ratio

## Discussion

Mutant ASXL1 is frequently present in different myeloid malignancies, namely MDS, myeloproliferative neoplasms (MPNs), MDS/ MPNs, and primary and secondary AML. In previous work by Chou WC et al., nearly ten percent of de-novo AML cases harbor ASXL1 mutations (25). Many researches have proposed the involvement of ASXL1 in epigenetic regulation (26-30). ASXL1 exhibits its involvement in epigenetic mechanisms by maintaining Polycomb Group (PcG) and Trithorax Group (trxG) proteins (8, 31). Noteworthy, ASXL1 mutations are present in clonal hematopoiesis of indeterminate potential (CHIP), proposing their link with early phases of leukemogenesis (32-34). 

Interestingly, ASXL1 may participate in normal adult hematopoiesis. However, its functions in embryonic hematopoiesis remain unclear (35). Due to the possible involvement of ASXL1 in hematopoiesis and the development of myeloid malignancies, it seems necessary to evaluate the impact of ASXL1 mutations on AML prognosis. Hence, we performed the current study to clarify the potential roles of ASXL1 mutations in the outcome of AML patients.

The influence of ASXL1 mutations on the outcome was assessed by comparing AML patients with mutated ASXL1 with those with wild-type ASXL1. Our findings demonstrate that ASXL1 mutations significantly reduce the OS of patients with AML in univariate and multivariate analyses (p<0.001). These findings parallel previous studies that proposed ASXL1 mutation as an adverse prognostic factor in other myeloid malignancies, such as MDS and CML (36, 37). Similarly, the subgroup analysis of the effects of ASXL1 mutations on RFS and EFS shows their significant influence on lower survival in patients with AML. We also analyzed the potentiality of ASXL1 mutations as prognostic markers in subgroups of AML cases presenting with intermediate-risk cytogenetic profiles and NPM1 mutations and intermediate-risk cytogenetic profiles without NPM1 mutations. However, we did not notice a meaningful correlation between ASXL1 mutations and the prognosis in these subgroups (p-value> 0.05). Noteworthy that great caution is necessary when interpreting these results mainly because of the extreme heterogeneity among included studies. Similarly, in a study by Chou et al. on 202 patients with intermediate-risk cytogenetics, they found no correlation between ASXL1 mutations and overall survival. However, the younger age of participants in this study (younger than 60 years) may be a possible explanation for this finding (25). 

On the contrary, in a paper by Devillier R et al., they proposed a significant relationship between ASXL1 mutations and the presence of intermediate-risk cytogenetics in patients with AML-MRC (38). A study by Ohgami et al. on 93 AML patients also showed that the prevalence of ASXL1 mutations in AML patients with myelodysplasia-related changes (AML-MRC) was higher than in patients with not otherwise specified therapy-related AML (AML-T, AML-NOS) and those with recurrent genetic abnormalities (AML-RGA) (39). Studies with larger sizes might present more detailed data regarding the prognostic effect of mutant ASXL1 co-occurrence with intermediate-risk cytogenetics or NPM1 mutations. 

Some investigations reported the co-occurrence of ASXL1 mutations with EZH2, IDH1/2, RUNX1, and TET2 in myeloid malignancies (40-42). As an illustration, Lin Y et al. suggested a relationship between ASXL1 and IDH1/2, NPM1, RUNX1, and TET2 mutations in patients with MDS (37). In addition, a study by Chou WC et al. in patients with primary AML proposed a close correlation between TET2 mutation and ASXL1 mutation (43, 44). Despite these findings, we could not perform a subgroup analysis for the prognostic effect of ASXL1 mutations in the presence of these mentioned mutations, mainly due to the need for more adequate data in the literature. A diverse range of mutations occurs in the ASXL1 gene, with frameshift c.1934dup and p. G646WfsX12 mutations more commonly observed in patients with AML. Of note, Paschka et al. identified c.1934dupG (p. G646WfsX12) as the most common ASXL1 mutation in patients with AML (11). Previous studies showed that frameshift and nonsense mutations are frequently found in the last exon of ASXL1, leading to C-terminal truncation of ASXL1 protein (29, 45, 46). Interestingly, Thol et al. (47) proposed that while frameshift mutations in ASXL1 were independent prognostic factors in MDS patients, point mutations were not independently associated with lower survival. However, due to the need for more data, we could not separately estimate the specific impact of each ASXL1 mutation on the prognosis of AML patients in the current meta-analysis. 

Moreover, some reports proposed the possible association between ASXL1 mutations and male sex (11, 18), older age (more than 65 years), and lower platelet counts in patients with myeloid malignancies (48, 49). However, these results could have been more consistent. While in the study of Paschka et al.’s, ASXL1 mutations were in significant association with increasing patients’ age (11), there was no statistically significant relationship in a study by Yu L et al. (P: 0.057) (50). In the current meta-analysis, subgroup analysis for any of these factors, including age, gender, or platelet count, was not conducted mainly because of insufficient data.

Furthermore, the majority of included studies (ten studies) in our meta-analysis used next-generation sequencing (NGS), eight papers applied direct Sanger sequencing, and three articles benefited both of these methods for detecting ASXL1 mutations. Studies have shown that NGS is a highly reliable tool for discovering gene mutations in AML patients (13, 51, 52). As an illustration, Yu G et al. (16) found that the NGS as a new platform has 100% sensitivity and specificity to discover important mutations, such as FLT3-ITD and c-Kit, compared to Sanger sequencing. In parallel, in a study by Duchmann et al. on patients with chronic myelomonocytic leukemia (CMML), they observed no significant difference between these two methods (53).

Even with our best attempts to perform a comprehensive meta-analysis, our study includes some limitations: 

1. The presence of additional genetic aberrations other than ASXL1 may result in heterogeneity in our meta-analysis.

2. Due to the need for additional data, we did not assess the impact of other possible sources of heterogeneity, such as the patient's demographic and clinical characteristics. Similarly, the type of therapies patients receive could affect their survival. 

3. While some studies reported multivariate HRs, others used univariate HRs, leading to heterogeneity. 

In conclusion, more comprehensive investigations with more participants from different populations and ethnicities are warranted to achieve a more persuasive conclusion.
